# Field Programmable Gate Array Based Parallel Strapdown Algorithm Design for Strapdown Inertial Navigation Systems

**DOI:** 10.3390/s110807993

**Published:** 2011-08-15

**Authors:** Zong-Tao Li, Tie-Jun Wu, Can-Long Lin, Long-Hua Ma

**Affiliations:** 1 Department of Control Science and Engineering, Zhejiang University, Hangzhou 310027, China; E-Mails: ztli@iipc.zju.edu.cn (Z.-T.L.); canlonglin@foxmail.com (C.-L.L.); 2 Institute of Navigation Guidance and Control, Zhejiang University, Hangzhou 310027, China; E-Mail: tjwu@zju.edu.cn (T.-J.W.)

**Keywords:** strapdown algorithm, coning and sculling compensation, parallelization design, computation complexity, FPGA

## Abstract

A new generalized optimum strapdown algorithm with coning and sculling compensation is presented, in which the position, velocity and attitude updating operations are carried out based on the single-speed structure in which all computations are executed at a single updating rate that is sufficiently high to accurately account for high frequency angular rate and acceleration rectification effects. Different from existing algorithms, the updating rates of the coning and sculling compensations are unrelated with the number of the gyro incremental angle samples and the number of the accelerometer incremental velocity samples. When the output sampling rate of inertial sensors remains constant, this algorithm allows increasing the updating rate of the coning and sculling compensation, yet with more numbers of gyro incremental angle and accelerometer incremental velocity in order to improve the accuracy of system. Then, in order to implement the new strapdown algorithm in a single FPGA chip, the parallelization of the algorithm is designed and its computational complexity is analyzed. The performance of the proposed parallel strapdown algorithm is tested on the Xilinx ISE 12.3 software platform and the FPGA device XC6VLX550T hardware platform on the basis of some fighter data. It is shown that this parallel strapdown algorithm on the FPGA platform can greatly decrease the execution time of algorithm to meet the real-time and high precision requirements of system on the high dynamic environment, relative to the existing implemented on the DSP platform.

## 1. Introduction

In a strapdown inertial navigation system (SINS), inertial sensors are rigidly attached to the vehicle, which leads to the system suffering from the highly dynamic vehicle movement environment. In addition, inertial sensors may be subject to high frequency motion as a result of body bending and engine-induced vibration. The strapdown algorithms adopted by most modern SINSs are constructed based on a general two-speed structure by which the position, velocity and attitude (PVA) updating operations are divided into two parts [1,2]: an exact moderate-speed updating routine (e.g., 50 ∼ 200 Hz) typically designed to update each PVA based on the maximum angular rate and acceleration of vehicle; and a high-speed updating routine (e.g., 1 ∼ 4 KHz for an aircraft INS with the positioning accuracy of less than 1 nmile/h) designed to accurately account for vibration-induced coning and sculling effects based on the anticipated vibration condition of the system. The original intention of the two-speed structure is to overcome the throughput limitations of early computer techniques, but the limitation is rapidly becoming insignificant with the continuous improvement in the performance of modern high-speed computers [3]. On the other hand, along with the fast progress of modern vehicles in ultra-high speed and other maneuvering performances, there exist more and more urgent demands to promote the navigation and control precision of the vehicles in high dynamic motion. It provides the motivation to return to a simpler single-speed structure of the strapdown algorithm in which all computations are executed at a single updating rate that is sufficiently high to accurately account for high frequency angular rate and acceleration rectification effects.

Two key compensation algorithms designed to operate in severe maneuvering and vibratory environments are critical in determining the performance of a SINS, *i.e.*, the coning compensation that works when the vehicle’s angular rate vector is rotating, and the sculling compensation that takes effect when the vehicle’s angular rate or specific force acceleration vector is rotating, or when the ratio of the angular rate to specific force is not constant. Thus in order to improve the navigation accuracy of the system, particularly in the environments where the angular rate vector or the specific force vector of the vehicle is large, several algorithms have been developed for the coning and sculling compensation. A substantial number of integration algorithms have been designed for coning compensation to improve the attitude accuracy without sacrificing computer throughput [4–11]. Analogous to the coning compensation algorithm adopted in attitude updating, a number of sculling compensation algorithms have also been designed for velocity updating, and the equivalence between coning and sculling compensation algorithms is discussed in [12,13]. A detailed statement of the coning and sculling compensation algorithms is given in [1,3,14–17]. Most algorithms for the coning and sculling compensations are based on truncated Taylor series expansion approximations for the angular rate of vehicle over updating cycles [2,3,6,7,9–11,18]. The accuracy of the coning and sculling compensation algorithms is determined by the updating rate of the coning and sculling compensations and the order of the truncated Taylor series expansion for the angular rate and specific force. Generally, in order to improve the accuracy of these algorithms, the updating rate must be increased to keep track of vehicle angular and linear motions more accurately. Among these existing algorithms, however, when the sampling rates of inertial sensors remain constant, and the number of the gyro incremental angle samples for coning compensation and the number of the accelerometer incremental velocity samples for sculling compensation are selected, the updating rates of these algorithms are also determined. The increase of the updating rates results in the decrease of the number of the gyro incremental angle samples and the number of the accelerometer incremental velocity samples for the coning and sculling compensation (namely, the decrease of order of the coning and sculling compensation algorithms), which in turn reduces the accuracy of the algorithms.

Furthermore, in recent SINS applications the strapdown algorithm with coning and sculling compensations is commonly implemented in a Digital Signal Processor (DSP) platform supplemented with Field Programmable Gate Array (FPGA) for data acquisition and noise filtering. Due to the serial execution mode of the DSP, however, it cannot support an updating rate fast enough for a high-order algorithm. In order to tackle the conflict between the computation complexity of the high-order algorithm and the updating speed of the algorithm implemented on a DSP platform, Xie [19] proposed a strapdown algorithm architecture on dual DSPs and FPGA which in essence works in a parallel computation mode. Jew [20] presented a framework for designing inertial navigation systems on a single-chip FPGA, in which the strapdown algorithm is implemented by the PowerPC hardcore of the FPGA. Although to some extent these methods improved the performance of the strapdown algorithms, they all work in a serial mode, thus it did not make full use of the parallel computation characteristics of the FPGA. Some other researchers [21,22] suggested using a single-chip FPGA to implement multi-processing cores and parallel computing, but there is no any implementation scheme discussed in detail.

In this paper, a new generalized optimum strapdown algorithm with coning and sculling compensations is presented in Section 2, in which the PVA updating operations are carried out based on the single-speed structure in which all computations are executed at a single updating rate that is sufficiently high to accurately account for high frequency angular rate and acceleration rectification effects. Different from existing algorithms, the updating rates of the coning and sculling compensations are unrelated with the number of the gyro incremental angle samples and the number of the accelerometer incremental velocity samples. Then, in order to implement the new strapdown algorithm in a single chip FPGA, the parallelization of the algorithm is designed in Section 3, and its computational complexity is analyzed in Section 4. In Section 5, the performance of the proposed parallel strapdown algorithm is tested on the software platform of Xilinx ISE 12.3 and the hardware platform of FPGA device XC6VLX550T on the basis of some fighter aircraft data. The contributions of this paper are finally summarized in Section 6.

## 2. Generalized Optimum Strapdown Algorithm

**Figure 1.**
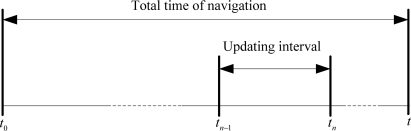
Intervals associated with strapdown algorithm.

In order to reduce the computational complexity and decouple the relationship between the updating rates of the coning and sculling compensations and the number of the gyro incremental angle and the accelerometer incremental velocity samples, the strapdown algorithm proposed in this section is constructed on the basis of a single-speed structure, *i.e*., the PVA are updated in all the intervals [*t_n_*_–1_,*t_n_*], *n* = 1, 2, …, with the equal length *T_n_* = *t_n_* – *t_n_*_–1_ in the whole navigation time [*t*_0_ ,*t*], as is shown in Figure 1.

According to the chain rule of matrix product, the updating of the attitude matrix $C_{B}^{N}$ is generally constructed as follows [1,2]:

(1) $$C_{B_{n}}^{N_{n}}=C_{N_{n-1}}^{N_{n}}C_{B_{n-1}}^{N_{n-1}}C_{B_{n}}^{B_{n-1}}$$

where $C_{B_{n-1}}^{N_{n-1}}$ and $C_{B_{n}}^{N_{n}}$ are the attitude matrixes relating the B frame to the N frame at time *t_n_*_–1_ and at time *t_n_*, respectively; $C_{B_{n}}^{B_{n-1}}$ is the direction cosine matrix that accounts for angular motion of the B frame from time *t_n_*_–1_ to time *t_n_*; $C_{N_{n-1}}^{N_{n}}$ is the direction cosine matrix that accounts for the N frame rotation frame from time *t_n_*_–1_ to time *t_n_*.

According to the velocity rate equation in the N frame [3], the velocity **v***^N^* at the time *t_n_* can be obtained by integrating the specific forces sensed by the accelerometers, the Coriolis accelerations due to the rotations of the navigation and earth frames and the gravity, namely:

(2) $$v_{n}^{N}=v_{n-1}^{N}+\Delta v_{{\mathrm{SF}}_{n}}^{N}+\Delta v_{{G/\mathrm{COR}}_{n}}^{N}$$

where $v_{n}^{N}$ and $v_{n-1}^{N}$ are the velocity of the system relative to the E frame at time *t_n_* and *t_n_*_–1_, respectively; $\Delta v_{{\mathrm{SF}}_{n}}^{N}$ and $\Delta v_{{G/\mathrm{COR}}_{n}}^{N}$ are the integrated transformed specific force increment and the gravity-Coriolis velocity increment over the updating interval [*t_n_*_–1_, *t_n_*], respectively, calculated by:

(3a) $$\Delta v_{{\mathrm{SF}}_{n}}^{N}=\int_{t_{n-1}}^{t_{n}}C_{B}^{N}f^{B}\mathrm{dt}$$

(3b) $$\Delta v_{{G/\mathrm{COR}}_{n}}^{N}=\int_{t_{n-1}}^{t_{n}}[g_{P}^{N}-\left(\omega_{\text{EN}}^{N}+2\omega_{\mathrm{IE}}^{N}\right)\times v^{N}]\mathrm{dt}$$

Considering the rotation of the navigation frame and the body frame over the updating interval [*t_n_*_–1_, *t_n_*], $\Delta v_{{\mathrm{SF}}_{n}}^{N}$ in Equation (3a) can be expanded according to the chain rule of matrix product as follows:

(4) $$\Delta v_{{\mathrm{SF}}_{n}}^{N}=\int_{t_{n-1}}^{t_{n}}C_{B}^{N}f^{B}dt=\int_{t_{n-1}}^{t_{n}}C_{N_{n-1}}^{N_{n}}C_{B_{n-1}}^{N_{n-1}}C_{B}^{B_{n-1}}f^{B}\mathrm{dt}=C_{N_{n-1}}^{N_{n}}C_{B_{n-1}}^{N_{n-1}}\Delta v_{{\mathrm{SF}}_{n}}^{B_{n-1}}$$

where:

(5) $$\Delta v_{{\mathrm{SF}}_{n}}^{B_{n-1}}=\int_{t_{n-1}}^{t_{n}}C_{B}^{B_{n-1}}f^{B}\mathrm{dt}$$

Because the variation of the position of the system is small over the updating interval [*t_n_*_–1_, *t_n_*] (for example, when the updating interval length *T_n_* is 0.0005 s, the variation of the position of the 6 Mach Hypersonic cruise missile is only approximately equal to 1.0 m), $g_{P}^{N}$ in Equation (3b) can be approximately by its mean value over the updating interval [*t_n_*_–1_, *t_n_*]. Similarly, the Coriolis term $\left(\omega_{\mathrm{EN}}^{N}+2\omega_{\mathrm{IE}}^{N}\right)\times v^{N}$ in Equation (3b) can also be approximately by its mean value over the updating interval [*t_n_*_–1_, *t_n_*] in view of the small variations of the angular rates $\omega_{\mathrm{IE}}^{N}$ and $\omega_{\mathrm{IE}}^{N}$ as well as the velocity **v***^N^* over the updating interval. Thus, the gravity-Coriolis velocity increment term $\Delta v_{{G/\mathrm{COR}}_{n}}^{N}$ can be approximated by:

(6) $$\begin{array}{ll} \Delta v_{{G/\mathrm{COR}}_{n}}^{N} & \approx \left\{g_{P_{n-1/2}}^{N}-\left[2\omega_{IE_{n-1/2}}^{N}+\omega_{EN_{n-1/2}}^{N}\right]\times v_{n-1/2}^{N}\right\}T_{n}\\ & =\left\{g_{P_{n-1/2}}^{N}-\left[2C_{E_{n-1/2}}^{N}\omega_{\mathrm{IE}}^{E}+F_{C_{n-1/2}}^{N}\left(u_{\mathrm{ZN}}^{N}\times v_{n-1/2}^{N}\right)+\rho_{ZN_{n-1/2}}u_{\mathrm{ZN}}^{N}\right]\times v_{n-1/2}^{N}\right\}T_{n} \end{array}$$

where $u_{\mathrm{ZN}}^{N}$ is the unit vector along the Z-axis of the navigation frame, and $u_{\mathrm{ZN}}^{N}={\left[\begin{array}{ccc} 0 & 0 & 1 \end{array}\right]}^{T}$.

According to the altitude and position matrix rate equation [3], the altitude *h* and position matrix $C_{E}^{N}$ at the time *t_n_* can be obtained as follows:

(7a) $$h_{n}=h_{n-1}+\Delta h_{n}$$

(7b) $$C_{E_{n}}^{N}=C_{N_{E_{n-1}}}^{N_{E_{n}}}C_{E_{n-1}}^{N}$$

where *h_n_* and *h_n_*_–1_ are the altitudes at the time *t_n_* and *t_n_*_–1_, respectively; Δ*h_n_* is the altitude change from the time *t_n_*_–1_ to the time *t_n_*; $C_{E_{n}}^{N}$ and $C_{E_{n-1}}^{N}$ are the position matrix at the time *t_n_* and *t_n_*_–1_, respectively; $C_{N_{E_{n-1}}}^{N_{E_{n}}}$ is the direction cosine matrix that accounts for the navigation frame rotation relative to the Earth frame from the time *t_n_*_–1_ to the time *t_n_*; and:

(8) $$\Delta h_{n}=\int_{t_{n-1}}^{t_{n}}u_{\mathrm{ZN}}^{N}v^{N}dt=u_{\mathrm{ZN}}^{N}\Delta R_{n}^{N}$$

Similar to the attitude matrix updating, $C_{N_{E_{n}}}^{N_{E_{n-1}}}$ in Equation (7b) can also be approximated in terms of a rotation vector as follows (accurate to second order):

(9) $$C_{N_{E_{n-1}}}^{N_{E_{n}}}\approx \text{I}-(\xi_{n}\times )+\frac{1}{2}(\xi_{n}\times )(\xi_{n}\times )$$

where ξ*_n_* is the rotation vector defining the navigation frame rotation relative to the earth frame from the time *t_n_*_–1_ to the time *t_n_*; and **ζ** *_n_* can be approximately expressed as follows:

(10) $$\xi_{n}\approx \int_{t_{n-1}}^{t_{n}}\omega_{\mathrm{EN}}^{N}dt=F_{C_{n-1/2}}^{N}\left(u_{\mathrm{ZN}}^{N}\times \Delta R_{n}^{N}\right)+\rho_{ZN_{n-1/2}}u_{\mathrm{ZN}}^{N}T_{n}$$

Note that $\Delta R_{n}^{N}$ should be calculated first to obtain the altitude *h* and the position matrix $C_{E}^{N}$ Considering that the change of the velocity is small over the updating interval [*t_n_*_–1_, *t_n_*], $\Delta R_{n}^{N}$ can be computed based on a trapezoidal integration algorithm as follows:

(11) $$\Delta R_{n}^{N}\approx \frac{1}{2}\left(v_{n}^{N}+v_{n-1}^{N}\right)T_{n}$$

### 2.1. Body Frame Rotation Update

The direction cosine matrix $C_{B_{n}}^{B_{n-1}}$ in Equation (1) is used to update the attitude matrix $C_{B}^{N}$ which accounts for the angular rate $\omega_{\mathrm{IB}}^{B}$ of the B frame relative to the inertial space. According to the relationship between rotation vector and direction cosine matrix, $C_{B_{n}}^{B_{n-1}}$ can be expressed as follows:

(12) $$C_{B_{n}}^{B_{n-1}}=I+\frac{\text{sin}\Phi_{n}}{\Phi_{n}}\left(\Phi_{n}\times \right)+\frac{1-\text{cos}\Phi_{n}}{\Phi_{n}^{2}}\left(\Phi_{n}\times \right)\left(\Phi_{n}\times \right)$$

where **Φ***n* is the rotation vector that accounts for angular motion of the body frame from time *t_n_*_–1_ to time *t_n_*; Φ*_n_* is the magnitude of **Φ***_n_* In practice, **Φ***_n_* can be obtained by the following rotation vector rate equation [4]:

(13) $$\Phi \approx \omega_{\mathrm{IB}}^{B}+\frac{1}{2}\Phi \times \omega_{\mathrm{IB}}^{B}+\frac{1}{12}\Phi \times \left(\Phi \times \omega_{\mathrm{IB}}^{B}\right)$$

where $\omega_{\mathrm{IB}}^{B}$ represents the angular rate of the B frame. The last two terms in Equation (13) are non-commutative, thus have to be calculated and compensated based on the gyro incremental angles in order to improve the computation accuracy. The triple-cross-product term in Equation (13) is usually quite small and can be neglected [4]. Then under the second order accuracy, the rotation vector **Φ***_n_* in Equation (12) can be approximated by the integral of Equation (13) from *t_n_*_–1_ to *t_n_*, *i.e*.,

(14) $$\Phi_{n}=\int_{t_{n-1}}^{t_{n}}\left[\omega_{\mathrm{IB}}^{B}+\frac{1}{2}\left(\alpha (t)\times \omega_{\mathrm{IB}}^{B}\right)\right]dt=\alpha_{n}+\beta_{n}$$

where **β** *_n_* is defined as the coning compensation from *t_n_*_–1_ to *t_n_*, and:

(15a) $$\alpha_{n}=\alpha (t_{n}),\;\alpha (t)=\int_{t_{n-1}}^{t}\omega_{\mathrm{IB}}^{B}d\tau$$

(15b) $$\beta_{n}=\beta (t_{n}),\;\;\beta (t)=\frac{1}{2}\int_{t_{n-1}}^{t}\left(\alpha (\tau )\times \omega_{\mathrm{IB}}^{B}\right)d\tau$$

For a SINS, the coning motion is the worst working condition which will induce serious attitude errors [5–7,18]. In other words, if in the case of coning movement the attitude errors are made minimal by a certain algorithm, the errors in the other cases will also be minimal by the same algorithm. So in order to develop the new strapdown algorithm, it is assumed that the vehicle is undergoing a pure coning movement, defined by the following angular rate:

(16) $$\omega_{\mathrm{IB}}^{B}(t)={\left[\alpha \Omega \text{cos}(\Omega t)\;\;b\Omega \text{sin}(\Omega t)\;\;0\right]}^{T}$$

where Ω is the frequency associated with the coning motion; *a* and *b* are the amplitudes of the coning motion.

According to Equations (15a), (15b) and (16), the coning compensation term **β***_n_* has the following form:

(17) $$\beta_{n}={\left[\begin{array}{ccc} 0 & 0 & \frac{\mathrm{ab}}{2}(\Omega T_{n}-\text{sin}\Omega T_{n}) \end{array}\right]}^{T}$$

Equation (17) shows an interesting property that the coning compensation is constant over all updating cycles, regardless of the absolute time at which the updating cycle begins. It only depends on the duration of the updating cycle.

According to the concept of distance between the cross products [6,11], the cross products with equal distance behave exactly the same in a pure coning environment defined by Equation (16). The coning compensation that uses the concept will have the simplest form, the optimal accuracy and the minimum computational throughput. Taking the advantage of this property, a generalized optimum algorithm for the integral in Equation (15b) consists of the sum of all possible cross products of the gyro incremental angle samples, making up the computation over the updating interval of rotation vector, such as [9]:

(18) $${\hat{\beta }}_{n}=\sum_{i=1}^{N-1}k_{i}\alpha_{n-i}\times \alpha_{n}$$

where *N* is the number of gyro incremental angle samples for the calculation of the coning compensation term; **α***_n–i_* (1, 2,…, *N* − 1) is the gyro incremental angle sample in the (*n* − *i*)-*th* updating cycle; *k_i_* (1, 2,…, *N* − 1) is the constant coefficients for the cross product of **α***_n–i_* and **α***_n_*.

Substituting Equations (15a) and (16) into Equation (18), and expanding each terms using Taylor series, the coning compensation term **β̂***_n_* over the updating interval [*t_n_*_–1_, *t_n_*] is obtained as:

(19) $${\hat{\beta }}_{n}={\left[\begin{array}{ccc} 0 & 0 & \mathrm{ab}\sum_{i=1}^{\infty }{\left(-1\right)}^{i+1}\sum_{j=1}^{N-1}A_{\mathrm{ij}}K_{j}\lambda^{2i+1} \end{array}\right]}^{T}$$

where *λ* = Ω*T_n_*; *A_ij_* is a constant defined by:

(20) $$A_{\mathrm{ij}}=\frac{{\left(j+1\right)}^{2i+1}-2•j^{2i+1}+{\left(j-1\right)}^{2i+1}}{(2i+1)!},i=1,2\cdots ,\infty ,j=1,2,\cdots ,N-1$$

In order to derive *k_i_* (1, 2,…, *N* − 1) in Equation (18), expanding Equation (17) by using Taylor series yields:

(21) $$\beta_{n}={\left[\begin{array}{ccc} 0 & 0 & \mathrm{ab}{\sum_{i=1}^{\infty }\left(-1\right)}^{i+1}C_{i}\lambda^{2i+1} \end{array}\right]}^{T}$$

where *C_i_* is a constant defined by:

(22) $$C_{i}=\frac{1}{(2i+1)!\times 2}$$

Combining Equation (19) with Equation (21), the following simultaneous equations for constant coefficients *K_i_*, *i* = 1, 2,…, *N* − 1), can be obtained:

(23) $$\sum_{j=1}^{N-1}A_{\mathrm{ij}}K_{j}=C_{i},i=1,2\cdots ,N-1$$

In a matrix form, Equation (23) is equivalent to:

(24) $${\left[A_{\mathrm{ij}}\right]}_{\left(N-1)\times (N-1\right)}\cdot {\left[K_{j}\right]}_{(N-1)\times 1}={\left[C_{i}\right]}_{(N-1)\times 1}$$

According to Equation (24), coefficients *K_i_* can be solved as follows:

(25) $${\left[K_{j}\right]}_{(N-1)\times 1}={\left[A_{\mathrm{ij}}\right]}_{\left(N-1)\times (N-1\right)}^{-1}\cdot {\left[C_{i}\right]}_{(N-1)\times 1}$$

where [*x_i_*]*_m_*_×1_ and [*x_ij_*]*_m_*_×1_ are *m*-dimensional column vector and the *m*-by-*n* matrix, respectively.

Note that different from other existing algorithms, the updating rate of the proposed optimal coning compensation algorithm is independent of the number of gyro incremental angle samples in the calculation of the coning compensation. Thus, this algorithm allows the updating speed to be increased, at the same time increasing the number of gyro incremental angle samples, in order to improve the attitude accuracy of the system.

### 2.2. Navigation Frame Rotation Update

The direction cosine matrix $C_{N_{n-1}}^{N_{n}}$ in Equation (1) is used to update the attitude matrix $C_{B}^{N}$ which accounts for the angular rate $\omega_{\mathrm{IN}}^{N}$ of the N frame relative to the inertial space. Similar to the computation of $C_{B_{n}}^{B_{n-1}}$, according to the relationship between rotation vector and direction cosine matrix, $C_{N_{n-1}}^{N_{n}}$ can also be expressed in a second order form as follows:

(25) $${\left[K_{j}\right]}_{(N-1)\times 1}={\left[A_{\mathrm{ij}}\right]}_{\left(N-1)\times (N-1\right)}^{-1}\cdot {\left[C_{i}\right]}_{(N-1)\times 1}$$

(26) $$C_{N_{n-1}}^{N_{n}}=\text{I}-\frac{\text{sin}\zeta_{n}}{\zeta_{n}}(\zeta_{n}\times )+\frac{1-\text{cos}\zeta_{n}}{\zeta_{n}^{2}}(\zeta_{n}\times )(\zeta_{n}\times )\approx \text{I}-(\zeta_{n}\times )+\frac{1}{2}(\zeta_{n}\times )(\zeta_{n}\times )$$

where **ζ** *_n_* is the rotation vector that accounts for the angular motion of the N frame from time *t_n_*_–1_ to time *t_n_*; **ζ** *_n_* is the magnitude of **ζ** *_n_* .

Because the updating interval length *T_n_* is short (generally equal to 0.0005 s–0.01 s), the angular rate $\omega_{\mathrm{IN}}^{N}$ is small and changes slowly over the updating interval [*t_n–1_*, *t_n_*] (due to the small changes in velocity and position over this updating cycle). Then according to the rotation vector rate equation, ζ*_n_* can be approximated as follows:

(27) $$\zeta_{n}\approx \int_{t_{n-1}}^{t_{n}}\omega_{\mathrm{IN}}^{N}dt=\int_{t_{n-1}}^{t_{n}}(C_{E}^{N}\omega_{\mathrm{IE}}^{E}+\omega_{\mathrm{EN}}^{N})dt\approx C_{E_{n-1/2}}^{N}\omega_{\mathrm{IE}}^{E}T_{n}+F_{C_{n-1/2}}^{N}\left(u_{\mathrm{ZN}}^{N}\times \Delta R_{n-1/2}^{N}\right)+\rho_{ZN_{n-1/2}}u_{\mathrm{ZN}}^{N}T_{n}$$

where $\omega_{\mathrm{IE}}^{E}$ and $\omega_{\mathrm{EN}}^{N}$ are the angular rates of the earth frame relative to the inertial frame and the navigation frame relative to the earth frame, respectively; $C_{B}^{N}$ is the position matrix relating the earth frame with the navigation frame; the subscript n − 1/2 indicates the midpoint of the updating interval [t*_n_*_–1_, t*_n_*].

### 2.3. Integrated Specific Force Increment Update

Similar to the attitude updating algorithm, the integral term $\Delta v_{{\mathrm{SF}}_{n}}^{B_{n-1}}$ in Equation (4) can be formulated based on the first order approximation of $C_{B}^{B_{n-1}}$ as follows:

(28) $$\Delta v_{{\mathrm{SF}}_{n}}^{B_{n-1}}\approx \int_{t_{n-1}}^{t_{n}}[\text{I}+(\alpha (t)\times ]f^{B}dt=\upsilon_{n}+\int_{t_{n-1}}^{t_{n}}(\alpha (t)\times f^{B})\mathrm{dt}$$

where:

(29) $$\upsilon_{n}=\upsilon (t_{n}),\;\;\;\;\upsilon (t)=\int_{t_{n-1}}^{t}f^{B}d\tau$$

The integrand term **α**(*t*)×**f***^B^* in Equation (28) has the following expression [3]:

(30) $$\alpha (t)\times f^{B}=\frac{1}{2}\frac{d}{\mathrm{dt}}(\alpha (t)\times \upsilon (t))+\frac{1}{2}(\alpha (t)\times f^{B}+\upsilon (t)\times \omega_{\mathrm{IB}}^{B})$$

$\Delta v_{SF_{n}}^{B_{n-1}}$ can also be expressed as follows:

(31) $$\Delta v_{SF_{n}}^{B_{n-1}}=\upsilon_{n}+\frac{1}{2}(\alpha_{n}\times \upsilon_{n})+\frac{1}{2}\int_{t_{n-1}}^{t_{n}}(\alpha (t)\times f^{B}+\upsilon (t)\times \omega_{\mathrm{IB}}^{B})dt=\upsilon_{n}+\Delta v_{Rot_{n}}+\Delta v_{Scul_{n}}$$

where Δ**v***_Rot_n__* denotes the velocity rotation compensation term; Δ**v***_Scult_n__* denotes the sculling compensation term; and:

(32a) $$\Delta v_{{\mathrm{Rot}}_{n}}=\frac{1}{2}(\alpha_{n}\times \upsilon_{n})$$

(32b) $$\Delta v_{{\mathrm{Scul}}_{n}}=\frac{1}{2}\int_{t_{n-1}}^{t_{n}}(\alpha (t)\times f^{B}+\upsilon (t)\times \omega_{\mathrm{IB}}^{B})\mathrm{dt}$$

In principle, the approaches used for the coning compensation can also be applied to the sculling compensation. Similar to the optimal generalized coning compensation algorithm in Equation (18), a generalized sculling compensation algorithm that has the simplest form, the optimal accuracy and the minimum computational throughput takes the following form:

(33) $$\Delta {\hat{v}}_{{\mathrm{Scul}}_{n}}=\left[\sum_{i=1}^{N-1}L_{i}\alpha_{n-i}\right]\times \upsilon_{n}+\left[\sum_{i=1}^{N-1}L_{i}\upsilon_{n-i}\right]\times \alpha_{n}$$

where *N* is the number of the gyro incremental angle samples and the number of the accelerometer incremental velocity samples for the calculation of the sculling compensation term; **α***_n–i_* (1, 2,…, *N* − 1) and **υ***_n–i_* (1, 2,…, *N* − 1) are the gyro incremental angle and accelerometer incremental velocity in the (*n* − *i*)*-th* updating cycle; *L_i_* (1, 2,…, *N* − 1) is the constant coefficients for the cross product of **α***_n–i_* with **υ***_n_* and **υ**_n–i_ with **α***_n_*.

Considering the equivalency between the coning compensation and sculling compensation [12,23], similar to Equation (25), the coefficients L*_i_* can also be calculated as follows:

(34) $${\left[L_{j}\right]}_{(N-1)\times 1}={\left[A_{\mathrm{ij}}\right]}_{\left(N-1)\times (N-1\right)}^{-1}\cdot {\left[C_{i}\right]}_{(N-1)\times 1}$$

where *A_ij_* and *C_i_* can be calculated according to Equations (20) and (22), respectively.

Note that different from other existing algorithms, the updating rate of the proposed optimal sculling compensation algorithm is also independent of the number of gyro incremental angle samples and accelerometer incremental velocity samples. Thus, this algorithm allows the updating speed to be increased, at the same time increasing the number of gyro incremental angle samples and accelerometer incremental velocity samples, in order to improve the accuracy of the related velocity updating algorithm.

### 2.4. Related Parameters Extrapolation Update

Because the gravity anomaly and the vertical deviation over the earth surface resulting from mass irregular and shape asymmetric distributions are generally small (the maximum value of the gravity anomaly is only tens of mgal; and the maximum value of the vertical deviation is only tens of arcs), the following approximate model of the gravity is used for most SINS applications:

(35) $$g(L,h)=g_{0}(L)\frac{R_{0}^{2}}{{\left(h+R_{0}\right)}^{2}}\approx g_{0}(L)\left(1-\frac{2h}{R_{0}}\right)$$

where $R_{0}=\sqrt{R_{M}R_{N}}$ and g_0_ is approximated by the following formula based on the WGS-84 data [24–27]:

(36) $$g_{0}(L)\approx 9.7803253359\frac{\left(1+0.001931853{\text{sin}}^{2}L\right)}{\sqrt{1-0.0066943{\text{sin}}^{2}L}}$$

Then the gravity $g_{P}^{N}$ in the navigation frame can be expressed as follows:

(37) $$g_{P}^{N}={\left[\begin{array}{ccc} 0 & 0 & -g(L,h) \end{array}\right]}^{T}$$

For the conventional ellipsoidal earth surface model and the E-N-U navigation frame [1,3], $\omega_{\mathrm{IE}}^{E}$, $\omega_{\mathrm{EN}}^{N}$ and $C_{E}^{N}$ can also be expressed in the following form:

(38a) $$\omega_{\mathrm{IE}}^{E}={\left[\begin{array}{ccc} 0 & 0 & \omega_{\mathrm{IE}} \end{array}\right]}^{T}$$

(38b) $$\omega_{\mathrm{EN}}^{E}=F_{C}^{N}(u_{\mathrm{ZN}}^{N}\times v^{N})+\rho_{\mathrm{ZN}}u_{\mathrm{ZN}}^{N}$$

(38c) $$\begin{array}{l} C_{E}^{N}=\left[\begin{array}{ccc} -\text{cos}\alpha \text{sin}\lambda -\text{sin}\alpha \text{sin}L\text{cos}\lambda & \text{cos}\alpha \text{cos}\lambda -\text{sin}\alpha \text{sin}L\text{sin}\lambda & \text{sin}\alpha \text{cos}L\\ \text{sin}\alpha \text{sin}\lambda -\text{cos}\alpha \text{sin}L\text{cos}\lambda & -\text{sin}\alpha \text{cos}\lambda -\text{cos}\alpha \text{sin}L\text{sin}\lambda & \text{cos}\alpha \text{cos}L\\ \text{cos}L\text{cos}\lambda & \text{cos}L\text{sin}\lambda & \text{sin}L \end{array}\right]\\ \;\;\;\;\;=\left[\begin{array}{ccc} C_{11} & C_{12} & C_{13}\\ C_{21} & C_{22} & C_{23}\\ C_{31} & C_{32} & C_{33} \end{array}\right] \end{array}$$

with:

(39a) $$F_{C}^{N}=\left[\begin{array}{ccc} \left(\frac{1}{R_{N}+h}-\frac{1}{R_{M}+h}\right)\text{sin}\alpha \text{cos}\alpha & -\left(\frac{{\text{sin}}^{2}\alpha }{R_{N}+h}+\frac{{\text{cos}}^{2}\alpha }{R_{M}+h}\right) & 0\\ \left(\frac{{\text{cos}}^{2}\alpha }{R_{N}+h}+\frac{{\text{sin}}^{2}\alpha }{R_{M}+h}\right) & \left(-\frac{1}{R_{N}+h}+\frac{1}{R_{M}+h}\right)\text{sin}\alpha \text{cos}\alpha & 0\\ 0 & 0 & 0 \end{array}\right]$$

(39b) $$\begin{array}{l} R_{M}=\frac{R_{e}{\left(1-e\right)}^{2}}{{\left[1-{\left(2-e\right)}^{2}{\text{sin}}^{2}L\right]}^{\frac{3}{2}}}\approx R_{e}(1+2e-3e{\text{sin}}^{2}L)\\ R_{N}=\frac{R_{e}}{{\left[1-(2-e)e{\text{sin}}^{2}L\right]}^{\frac{1}{2}}}\approx R_{e}\left(1+e{\text{sin}}^{2}L\right) \end{array}$$

(39c) $${\text{sin}}^{2}L={\left(C_{33}\right)}^{2},\text{sin}\alpha \text{cos}\alpha =\frac{C_{13}C_{23}}{1-{\left(C_{33}\right)}^{2}},{\text{sin}}^{2}\alpha =\frac{{\left(C_{13}\right)}^{2}}{1-{\left(C_{33}\right)}^{2}},{\text{cos}}^{2}\alpha =\frac{{\left(C_{23}\right)}^{2}}{1-{\left(C_{33}\right)}^{2}}$$

where $F_{C}^{N}$ is the curvature matrix in the navigation frame that is a function of position over the earth; ρ*_ZN_* is the Z-axis component of $\omega_{\mathrm{EN}}^{N}$; **v***^N^* is the velocity vector of the vehicle relative to the earth projected on the navigation frame; *R_M_* and *R_N_* are the radii of curvature at the earth surface in meridian and in prime vertical, respectively; *h* is the height; *α* is the wander angle; *L* is the latitude; *R_e_* is the equatorial radius of earth; *e* is the flattening of earth; *C_ij_* is the *i*-row and *j*-column component of $C_{E}^{N}$.

By the definition of the navigation frame, the orientation of the X axis and the Y axis around the Z axis is somewhat arbitrary. So *ρ_ZN_* in Equation (38b) depends on the selection of the axes orientation of the navigation frame. The navigation frame is generally selected as a wander azimuth navigation frame for most SINSs [3]. In this case, *ρ_ZN_* is given by:

(40) $$\rho_{\mathrm{ZN}}=0$$

Because the related parameters ( ) (namely, $g_{P}^{N}$, $F_{C}^{N}$, ρ*_ZN_*, **v***^N^*, Δ**R***^N^* and $C_{E}^{N}$) in Equations (6), (10) and (27) are all the functions of position or velocity, and the values of these parameters at the current cycle are not available, thus the ( )_n − 1/2_ terms can be approximately calculated based on the linear extrapolation formula in the following form:

(41) $${\left(\;\right)}_{n-1/2}\approx {\left(\;\right)}_{n-1}+\frac{1}{2}[{\left(\;\right)}_{n-1}-{\left(\;\right)}_{n-2}]=\frac{3}{2}{\left(\;\right)}_{n-1}-\frac{1}{2}{\left(\;\right)}_{n-2}$$

## 3. Strapdown Algorithm Parallelization

Although the sampling rate of inertial sensors can be up to 2 kHz or even higher, taking into account the complexity of the employed strapdown algorithm and the ability of the current processors, the updating rate of the strapdown algorithm in a serial mode is limited, usually only 200–500 Hz when implemented in a DSP. An effective way to break through the limitation of commonly used navigation computers is to implement the strapdown algorithm on a purely parallel computing platform such as FPGA, and execute the calculations in the algorithm “as concurrently as possible” to make full use of the capability of the parallel computing platform.

**Figure 2.**
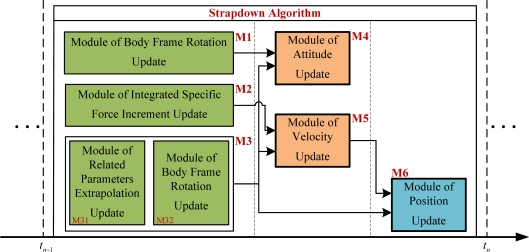
Functional-block diagram of parallel strapdown algorithm.

The strapdown algorithm proposed in Section 2 can be divided into six modules doing the following calculations severally: the body frame rotation update (M1), the integrated specific force increment update (M2), the related parameters extrapolation update and the navigation frame rotation update (M3), the attitude update (M4), the velocity update (M5) and the position update (M6), as shown in Figure 2. Among them, M1, M2 and M3 can be executed first in a parallel mode; M4 and M5 have to be executed afterwards but also in a parallel mode; finally, M6 is executed.

### 3.1. Module of Body Frame Rotation Update (M1)

**Figure 3.**
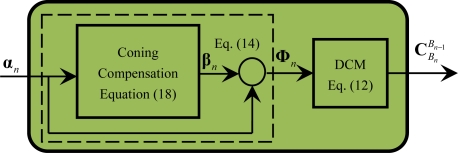
Diagram of module M1.

The calculations involved in the module M1 are described by Equations (12), (14) and (18), as shown in Figure 3. Once the number of the gyro incremental angle samples *N* is selected, the corresponding coefficients *K_i_* of the coning compensation can firstly be determined offline by Equations (20), (22) and (25) and stored in the memory of the navigation computer for online use; secondly, the coning compensation **β***_n_* and rotation vector **Φ***_n_* can be successively computed according to Equations (14) and (18), respectively; finally, the direction cosine matrix $C_{B_{n}}^{B_{n-1}}$ that accounts for the angular motion of the B frame is obtained by Equation (12). Note that since the accuracy of the coning compensation directly determines the attitude accuracy of the system, particularly in high dynamic conditions, the coning compensation is generally designed to accurately account for the vibration induced coning effects by selecting the appropriate number of the gyro incremental angle samples.

Figure 3 shows that Equations (11), (14) and (18) can only be executed in a serial mode. From Equations (12), (14) and (18), it is shown that M1 contains the following operations of related minimum calculation particles: cross-product of vectors, product of a skew symmetric matrix with itself, addition of matrixes or vectors and calculation of sine or cosine function. The operations within these minimum calculation particles can be further concurrently processed, which will be discussed in Section 3.2.

### 3.2. Module of Integrated Specific Force Increment Update (M2)

**Figure 4.**
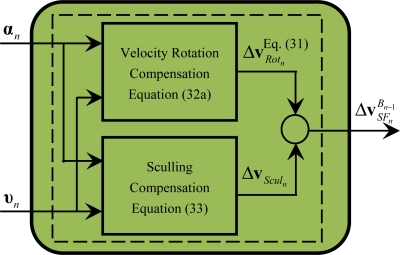
Diagram of module M2.

The module M2 carries out the calculations defined by Equations (31), (32a) and (33), as shown in Figure 4. Similar to the coning compensation, once the number of the gyro incremental angle and accelerometer incremental velocity samples *N* is selected, the corresponding coefficients *L_i_* of the sculling compensation can firstly be determined offline by Equations (20), (22) and (34) and stored in the memory of the navigation computer for online use; secondly, the sculling compensation Δ**v***_Scul_n__* and the velocity rotation compensation Δ**v***_Rot_n__* can be successively computed according to Equations (32a) and (33); finally, the integrated specific force increment $\Delta v_{{\mathrm{SF}}_{n}}^{B_{n-1}}$ that accounts for the linear motion of the B frame is calculated by Equation (31). Note that since the accuracy of the sculling compensation directly determines the velocity accuracy of the system, particularly in high dynamic conditions, the sculling compensation is generally designed to accurately account for the vibration induced sculling effects by selecting the appropriate number of the gyro incremental angle and accelerometer incremental velocity samples.

Figure 4 shows that the calculations of the velocity rotation compensation and the sculling compensation can be executed in a parallel mode according to Equations (32a) and (33). From Equations (31), (32a) and (33), it is shown that M2 contains the following operations of related minimum calculation particles: cross-product of vectors and addition of vectors. Similar to the operations within these minimum calculation particles in M1, the operations within these minimum calculation particles can be further processed concurrently, which will be discussed in Section 3.2.

### 3.3. Module of Related Parameters Extrapolation and Navigation Frame Rotation Update (M3)

**Figure 5.**
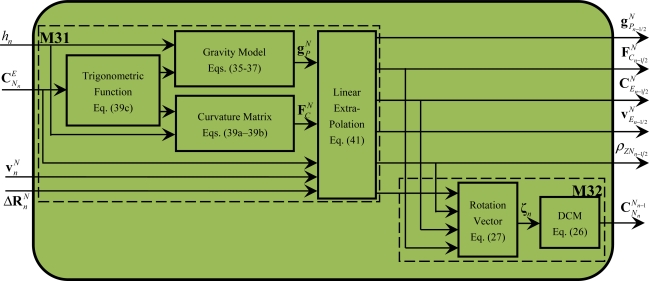
Diagram of module M3.

The module M3 can be further divided into two serial-process modules: the module of related parameters extrapolation update (M31) and the module of navigation frame rotation update (M32), as shown in Figure 5. The computation tasks completed in M31 and M32 are described by Equations (26), (27) and (35–41) respectively.

Figure 5 shows that in M31, the calculation of the gravity and the curvature matrix can be firstly executed in a parallel mode according to Equations (35–39), and then the related parameters ( $g_{P_{n-1/2}}^{N}$, $v_{E_{n-1/2}}^{N}$, $F_{C_{n-1/2}}^{N}$, ρ*_ZN_n–1/2__*, $\Delta R_{n-1/2}^{N}$ and $C_{E_{n-1/2}}^{N}$) can also be calculated in a parallel mode according to Equation (41); in M32, Equations (26) and (27) can only be executed in a serial mode. From Equations (26), (27) and Equations (35–41), it is shown that M31 and M32 contain the following operations of related minimum calculation particles: cross-product of vectors, product of a skew symmetric matrix with its own and addition of matrixes or vectors. Similar to the operations within these minimum calculation particles in module M1, the operations within these minimum calculation particles can be further processed concurrently, which will be discussed in Section 3.2.

### 3.4. Module of Attitude Update (M4)

**Figure 6.**
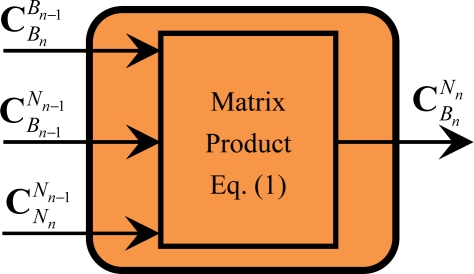
Diagram of module M4.

The module M4 is used to calculate Equation (1), as shown in Figure 6.

From Equation (1), it is shown that M4 only contains the following operation of related minimum calculation particles: product of matrixes. Similar to the operations within these minimum calculation particles in module M1, the product operation of matrixes can be further processed concurrently, which will be discussed in Section 3.2.

### 3.5. Module of Velocity Update (M5)

**Figure 7.**
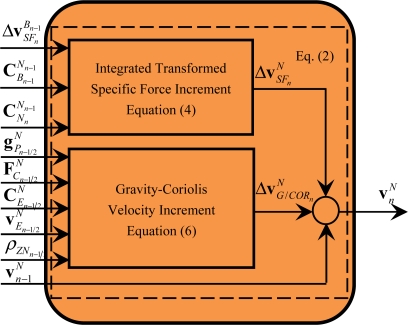
Diagram of module M5.

The calculations implemented in the module M5 are defined by Equations (2), (4) and (6), as shown in Figure 7.

Figure 7 shows that the calculation of the integrated transformed specific force increment and the gravity-Coriolis velocity increment can be executed in a parallel mode according to Equations (4) and (6). From Equations (2), (4) and (6), it is shown that M5 contains the following operations of related minimum calculation particles: cross-product of vectors, product of a skew symmetric matrix with its own, product of matrixes or a matrix with a vector and addition of vectors. Similar to the operations within these minimum calculation particles in module M1, the operations within these minimum calculation particles can be further parallelized, which will be discussed in Section 3.2.

### 3.6. Module of Position Update (M6)

**Figure 8.**
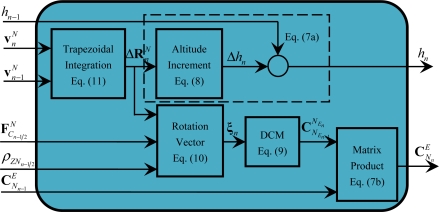
Diagram of module M6.

In the module M6, the computations defined by Equations (7–11) are carried out, as shown in Figure 8.

Figure 8 shows that after the calculation of $\Delta R_{n}^{N}$, the calculation of the altitude *h* and the position matrix $C_{E}^{N}$ can be executed in a parallel mode according to Equations (7–10), in which Equations (7b), (9) and (10) can only be executed in a serial mode, and Equations (7a) and (8) can also only be executed in a serial mode. From Equations (7–11), it is shown that M6 contains the following operations of related minimum calculation particles: cross-product of vectors, product of a matrix with a vector and addition of vectors. Similar to the operations within these minimum calculation particles in module M1, the operations within these minimum calculation particles can be further parallelized, which will be discussed in Section 3.2.

## 4. Computation Complexity Analysis

From Figure 2, it can be shown that the execution time of the parallel strapdown algorithm on FPGA can be as follows:

(42) $$T_{\mathrm{Nov}}=\text{max}\{T_{M1},T_{M2},T_{M3}\}+\text{max}\{T_{M4},T_{M5}\}+T_{M6}$$

with:

(43) $$T_{M3}=T_{M31}+T_{M32}$$

where, *T_M_*_1_, *T_M_*_2_, *T_M_*_3_, *T_M_*_4_, *T_M_*_5_ and *T_M_*_6_ are the execution time required by the modules M1, M2, M3, M4, M5 and M6, respectively; *T_M_*_31_ and *T_M_*_32_ are the execution time required by the modules M31 and M32, respectively.

In order to make the updating cycle of the strapdown algorithm shortest, the maximum parallelism degree is usually used as a performance index to optimize the calculation particles involved in the modules M1–M6.

Assume that the execution time of addition (subtraction), multiplication, division, trigonometric and square root operation are defined as *T_A_*, *T_M_*, *T_D_*, *T_T_* and *T_S_*, respectively. The summation of 3-dimensional vectors, for instance by:

(44) $$v_{\mathrm{VA}}=v_{1}+v_{2}$$

contains three addition operations which can be executed in a parallel mode. Thus the execution time of the addition operation for two 3-dimensional vectors is

(45) $$T_{\mathrm{VA}}=T_{A}$$

The addition of two 3-by-3 matrixes, for instance by:

(46) $$C_{\mathrm{MA}}=C_{1}+C_{2}$$

contains nine addition operations which can also be executed in a parallel mode. Thus the execution time of the addition operation for two 3-by-3 matrixes is

(47) $$T_{\mathrm{MA}}=T_{A}$$

The cross-product of two 3-dimensional vectors, expressed for instance by:

(48) $$v_{\mathrm{VCP}}=v_{1}⊗v_{2}=\left[\begin{array}{ccc} 0 & -V_{1Z} & V_{1Y}\\ V_{1Z} & 0 & -V_{1X}\\ -V_{1Y} & V_{1X} & 0 \end{array}\right]\left[\begin{array}{c} V_{2X}\\ V_{2Y}\\ V_{2Z} \end{array}\right]=\left[\begin{array}{c} V_{1Y}V_{2Y}-V_{1Z}V_{2X}\\ V_{1Z}V_{2X}-V_{1X}V_{2Z}\\ V_{1X}V_{2Y}-V_{1Y}V_{2X} \end{array}\right]$$

contains six multiplication operations and three subtraction operations. All the multiplication operations or the subtraction operations can be executed in a parallel mode, but the subtraction operations must be executed after the multiplication operations. Thus the execution time of the cross-product operation for two 3-dimensional vectors is:

(49) $$T_{\mathrm{VCP}}=T_{M}+T_{A}$$

The product of two 3-by-3 matrixes, for instance by:

(50) $$C_{C}^{A}=C_{B}^{A}C_{C}^{B}$$

contains 27 multiplication operations and 18 addition operations. The multiplication operations can be executed in a parallel mode, but the addition operations must be executed twice in a parallel mode after the multiplication operations. Thus the execution time of the product operation for two 3-by-3 matrixes is:

(51) $$T_{\mathrm{MP}}=T_{M}+2T_{A}$$

The product of a 3-by-3 matrix with a 3-dimensional vector, which can be defined for instance by:

(52) $${\text{V}}^{A}=C_{B}^{A}{\text{V}}^{B}$$

contains nine multiplication operations and 6 addition operations. The multiplication operations can be executed in a parallel mode, but the addition operations must be executed twice in a parallel mode after the multiplication operations. Thus the execution time of the product operation for a 3-by-3 matrix with a 3-dimensional vector is:

(53) $$T_{\mathrm{MVP}}=T_{M}+2T_{A}$$

The product of a skew symmetric matrix with itself defined, for instance by:

(54) $$\begin{array}{l} C=({\text{V}}_{1}⊗)({\text{V}}_{1}⊗)\\ \;\;\;=\left[\begin{array}{ccc} 0 & -V_{1Z} & V_{1Y}\\ V_{1Z} & 0 & -V_{1X}\\ -V_{1Y} & V_{1X} & 0 \end{array}\right]\left[\begin{array}{ccc} 0 & -V_{1Z} & V_{1Y}\\ V_{1Z} & 0 & -V_{1X}\\ -V_{1Y} & V_{1X} & 0 \end{array}\right]\left[\begin{array}{ccc} -V_{1Z}^{2}-V_{1Y}^{2} & V_{1X}V_{1Y} & V_{1X}V_{1Z}\\ V_{1X}V_{1Y} & -V_{1Z}^{2}-V_{1X}^{2} & V_{1Y}V_{1Z}\\ V_{1X}V_{1Z} & V_{1Y}V_{1Z} & -V_{1Y}^{2}-V_{1X}^{2} \end{array}\right] \end{array}$$

contains six multiplication operations and three addition operations. The multiplication operations or the addition operations can be executed in a parallel mode, but the addition operations must be executed after the multiplication operations. Thus the execution time of the product operation for a skew symmetric matrix with itself is:

(55) $$T_{\mathrm{VCP}2}=T_{M}+T_{A}$$

Based on the aforementioned execution time analysis of the basic computational operations involved in the modules M1–M6, we can evaluate the computational complexity of each module.

### 4.1. Analysis of Module M1

In the calculation of the coning compensation term **β***_n_* defined by Equation (18), the *N* − 1 vectors cross-product operation can be executed in a parallel mode, and the summation of *N* − 1 vectors can also be successively executed in a parallel mode [log_2_(N – 1)] times. Thus the execution time of the coning compensation is:

(56) $$T_{\mathrm{Con}}=T_{\mathrm{VCP}}+T_{M}+\left\lceil {\text{log}}_{2}(N-1)\right\rceil T_{\mathrm{VA}}=2T_{M}+\left[\left\lceil {\text{log}}_{2}(N-1)\right\rceil +1\right]T_{A}$$

And in the calculation of the direction cosine matrix $C_{B_{n}}^{B_{n-1}}$ according to Equation (12), the calculation of $\Phi_{n}^{2}$, **Φ***_n_* and (**Φ***_n_* ×) (**Φ***_n_* ×) can be executed in a parallel mode. Thus the execution time of the matrix $C_{B_{n}}^{B_{n-1}}$ calculation is:

(57) $$T_{\mathrm{Rot}2\mathrm{DCM}}=T_{M}+2T_{A}+T_{S}+T_{T}+T_{A}+T_{D}+T_{M}+2T_{A}=2T_{M}+4T_{A}+T_{D}+T_{S}+T_{T}$$

Based on Equations (56) and (57) and refer to Figure 3, the execution time of module M1 can be obtained as follows:

(58) $$T_{M1}=T_{\mathrm{Con}}+T_{\mathrm{VA}}+T_{\mathrm{Rot}2\mathrm{DCM}}=4T_{M}+\left[\left\lceil {\text{log}}_{2}(N-1)\right\rceil +6\right]T_{A}+T_{D}+T_{S}+T_{T}$$

### 4.2. Analysis of Module M2

Figure 5 shows that the calculation of the velocity rotation compensation and the sculling compensation can be executed in a parallel mode. According to Equation (48), the execution time of the velocity rotation compensation is:

(59) $$T_{\mathrm{Rot}}=T_{\mathrm{VCP}}+T_{M}=2T_{M}+T_{A}$$

Similar to the calculation of the coning compensation term **β***_n_*, in the calculation of the sculling compensation term Δ**v**_*Scul*_*n*__ defined by Equation (33), the cross-product operations of the 2(*N* – 1) vectors can be executed in a parallel mode, and the summation of *N* − 1 vectors can also be successively executed in a parallel mode [log_2_(*N* – 1)] times. Thus the execution time of the coning compensation is:

(60) $$T_{\mathrm{scul}}=T_{\mathrm{VCP}}+T_{M}+\left\lceil {\text{log}}_{2}(N-1)\right\rceil T_{\mathrm{VA}}=2T_{M}+\left[\left\lceil {\text{log}}_{2}(N-1)\right\rceil +1\right]T_{A}$$

Based on Equations (59) and (60) and referring to Figure 4, the execution time of module M2 can be obtained as follows:

(61) $$T_{M2}=\text{max}\{T_{\mathrm{Rot}},T_{\mathrm{scul}}\}+2T_{\mathrm{VA}}=2T_{M}+\left[\left\lceil {\text{log}}_{2}(N-1)\right\rceil +3\right]T_{A}$$

### 4.3. Analysis of Module M3

Figure 6 shows that the calculation of the gravity and the curvature matrix can be executed in a parallel mode. According to Equations (35–37), the execution time of the gravity calculation is:

(62) $$T_{G}=\text{max}\{T_{G1},T_{G2},T_{G3}\}+2T_{M}+T_{D}$$

where *T_G_*_1_, *T_G_*_2_ and *T_G_*_3_ are the execution time of the calculations for the terms (1+0.001931853sin^2^ *L*), $\sqrt{1-0.006694380{\text{sin}}^{2}L}$ and $\left(1-\frac{2h}{R_{0}}\right)$, respectively; and:

(63a) $$T_{G1}=T_{M}+T_{A}$$

(63b) $$T_{G2}=T_{M}+T_{A}+T_{S}$$

(63c) $$T_{G3}=T_{M}+T_{A}+T_{D}$$

According to Equation (39), the execution time of the curvature matrix calculation is:

(64) $$T_{F}=\text{max}\{T_{F1},T_{F2}\}+T_{A}+T_{D}+2T_{M}+T_{A}$$

where *T_F_*_1_ and *T_F_*_1_ are the execution time of the calculations for *R_M_* and *R_N_*, respectively; and:

(65a) $$T_{F1}=3T_{M}+2T_{A}$$

(65b) $$T_{F2}=2T_{M}+T_{A}$$

Thus the execution time of module M31 can be obtained as follows:

(66) $$T_{M31}=T_{M}+T_{A}+T_{M}+T_{D}+\text{max}\{T_{G},T_{F}\}+T_{M}+T_{A}$$

According to Equations (26) and (27), the execution time of module M32 can be estimated by:

(67) $$T_{M32}=(T_{M}+T_{\mathrm{MVP}}+T_{A})+(T_{\mathrm{VCP}2}+T_{M}+T_{A})=4T_{M}+5T_{A}$$

Based on Equations (66) and (67), and refer to Figure 6, the total execution time of module M3 is:

(68) $$T_{M3}=T_{M31}+T_{M32}$$

### 4.4. Analysis of Module M4

According to Equation (1), the execution time of module M4 is as follows:

(69) $$T_{M4}=2T_{\mathrm{MP}}=2T_{M}+4T_{A}$$

### 4.5. Analysis of Module M5

Figure 8 shows that the calculation of the integrated transformed specific force increment and the gravity-Coriolis velocity increment can be executed in a parallel mode. According to Equation (4), the execution time of the integrated transformed specific force increment calculation is:

(70) $$T_{\mathrm{SF}}=T_{\mathrm{MP}}+T_{\mathrm{MVP}}=2T_{M}+4T_{A}$$

According to Equation (6), the execution time of the gravity-Coriolis velocity increment calculation is:

(71) $$T_{G-C}=T_{\mathrm{MVP}}+T_{M}+T_{A}+T_{\mathrm{VCP}}+T_{A}+T_{M}=4T_{M}+5T_{A}$$

Based on Equations (70) and (71), and refer to Figure 8, the execution time of module M5 can be obtained as follows:

(72) $$T_{M5}=\text{max}\{T_{\mathrm{SF}},T_{G-C}\}+2T_{A}=4T_{M}+7T_{A}$$

### 4.6. Analysis of Module M6

**Figure 9.**
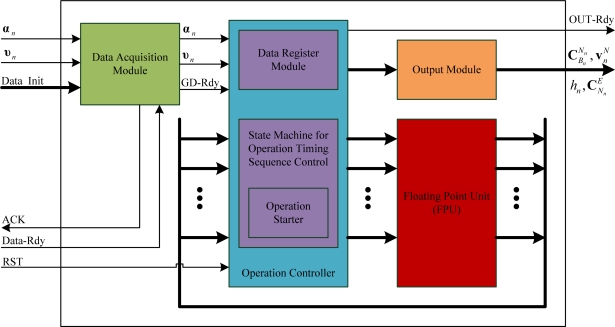
Implementation of parallel strapdown algorithm base on FPGA.

Figure 9 shows that the calculation of the altitude matrix and the position matrix can be executed in a parallel mode. According to Equations (7a), (8) and (11), the execution time of the altitude calculation is:

(73) $$T_{\mathrm{Alt}}=T_{\mathrm{VA}}+2T_{M}+T_{A}=2T_{M}+2T_{A}$$

According to Equations (7b) and Equations (9–11), the execution time of the position matrix calculation is:

(74) $$T_{\mathrm{PosDCM}}=T_{\mathrm{VA}}+2T_{M}+T_{M}+T_{A}+T_{\mathrm{VCP}2}+T_{M}+T_{A}+T_{\mathrm{MP}}=6T_{M}+6T_{A}$$

Based on Equations (73) and (74) and refer to Figure 9, the execution time of module M6 can be obtained as follows:

(75) $$T_{M6}=\text{max}\{T_{\mathrm{Alt}},T_{\mathrm{PosDCM}}\}=6T_{M}+6T_{A}$$

The execution times of the floating-point operations *T_A_*, *T_M_*, *T_D_*, *T_T_* and *T_S_* are slightly different when implemented on different FPGA platforms. But compared with *T_m_*, *T_A_* and *T_D_*, *T_T_* and *T_S_* are generally larger and has the following approximate relationship with *T_M_*:

(76) $$T_{D}\approx 4T_{M},\;\;T_{T}\approx 4T_{M},\;\;\;T_{S}\approx 4T_{M},\;\;\;T_{A}\approx 2T_{M}$$

And the number of the gyro incremental angle samples and the accelerometer incremental velocity samples used in the calculation of the coning compensation and the sculling compensation is generally less than four [2], namely, N ≤ 4.Then according to Equations (42), (58), (61), (68), (69), (72) and (75), the length of the execution time of the parallelized strapdown algorithm can be estimated as follows:

(77) $$\begin{array}{ll} T_{\mathrm{Nav}} & =\text{max}\{T_{M1},T_{M2},T_{M3}\}+\text{max}\{T_{M4},T_{M5}\}+T_{M6}\\ & =T_{M3}+T_{M5}+T_{M6}=22T_{M}+24T_{A}+2T_{D}\approx 78T_{M} \end{array}$$

In contrast with the parallel implementation of the strapdown algorithm proposed in Section 3, the execution time of the original strapdown algorithm in a serial mode given in Section 2 is:

(78) $$T_{\mathrm{Nav}}^{\prime }=T_{M1}^{\prime }+T_{M2}^{\prime }+T_{M3}^{\prime }+T_{M4}^{\prime }+T_{M5}^{\prime }+T_{M6}^{\prime }$$

with:

(79a) $$T_{M1}^{\prime }=(7N+11)T_{M}+(4N+16)T_{A}+2T_{D}+T_{S}+2T_{T}$$

(79b) $$T_{M2}^{\prime }=(14N-5)T_{M}+(8N-1)T_{A}$$

(79c) $$T_{M3}^{\prime }=88T_{M}+57T_{A}+7T_{D}+T_{S}$$

(79d) $$T_{M4}^{\prime }=54T_{M}+36T_{A}$$

(79e) $$T_{M5}^{\prime }=43T_{M}+35T_{A}$$

(79f) $$T_{M6}^{\prime }=49T_{M}+34T_{A}$$

Thus according to Equations (76–79), the ratio of the execution time of the parallelized strapdown algorithm to the execution time of the original strapdown algorithm can be estimated as follows:

(80) $$\eta =\frac{T_{\mathrm{Nav}}}{T_{\mathrm{Nav}}^{\prime }}=\frac{22T_{M}+24T_{A}+2T_{D}}{(240+21N)T_{M}+(177+12N)T_{A}+9T_{D}+2T_{S}+2T_{T}}\approx 9.44\%$$

where *N* is assumed to be 4. Equation (80) indicate that the parallelization design of the new optimum strapdown algorithm can significantly increase the updating rate of the algorithm, thus providing an important foundation to improve the accuracy of SINS working in high dynamic environments.

## 5. Implementation and Simulation of Parallel Strapdown Algorithm on FPGA

The parallel strapdown algorithm proposed in Section 3 has been implemented on a FPGA platform in the structure shown in Figure 9. The data acquisition module receives the input signal (the gyro incremental angle **α***_n_*, the accelerometer incremental velocity **υ***_n_* and the initial alignment data *Data_Init*) and writes the data to the data register module in the operation controller, then notifies the operation controller to start the strapdown calculations through the signal *GD_Rdy*. The operation controller sends the data stored in the data register module in a parallel mode to the input registers of the floating point unit (FPU), and starts the FPU by the operation starter. The operation results ( $C_{B_{n}}^{N_{n}}$, $v_{n}^{N}$, *h_n_* and $C_{N_{n}}^{E}$) are then exported through the output module. The handshake signals *ACK* and *Data_Rdy* are used for the communication between the data acquisition module and the external module such as the initial alignment or noise filtering of inertial sensor output samples that is beyond the scope of this paper; and the operation controller can be reset by the signal *RST*. The state machine in the operation controller is used to control the execution of operations in an appropriate time sequence.

All floating-point operations are carried out in the FPU which is composed of five arithmetic sub-units executing the operations for addition, multiplication, division, square root calculation and trigonometric calculation, respectively. Among them, the adder unit contains *k* floating-point adders; the multiplier unit contains *l* floating-point adders; the divider unit contains *m* floating-point adders; the square root arithmetic unit contains *n* floating-point adders; where different values of *k*, *l*, *m* and *n* can be selected according to the hardware resources of the selected FPGA platform.

The parallel strapdown algorithm has been simulated on the Xilinx ISE 12.3 software platform and the hardware platform of the FPGA device XC6VLX550T. The floating-point adder/subtractor, multiplier and other floating-point operations in FPU are constructed by the Xilinx IP core.

In the simulation, both the number of the gyro incremental angle samples and the number of the accelerometer incremental velocity samples for the calculation of the coning compensation and the sculling compensation are set to two (namely, *N* = 2), then, according to Equations (20), (22), (25) and (34), the coning compensation term and the sculling compensation term defined in Equations (18) and (33) can be expressed as follows:

(81a) $${\hat{\beta }}_{n}=\frac{1}{12}\alpha_{n-1}\times \alpha_{n}$$

(81b) $$\Delta {\hat{v}}_{{\mathrm{Scul}}_{n}}=\frac{1}{12}\alpha_{n-1}\times \upsilon_{n}+\frac{1}{12}\upsilon_{n-1}\times \alpha_{n}$$

**Figure 10.**
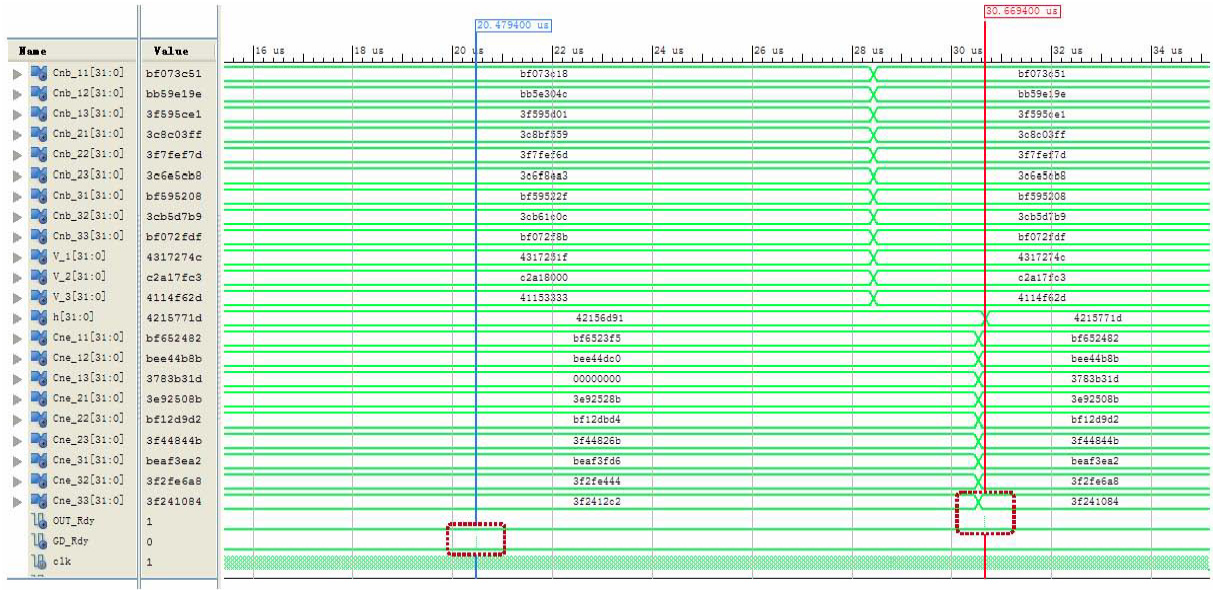
Behavioral simulation waveform graph of parallel strapdown algorithm.

To demonstrate the performance of the proposed parallel strapdown algorithm, the simulation results in a typical updating interval are shown in Figure 10 as the behavioral simulation waveform graph yielded by Xilinx ISE 12.3, and listed in Table 1 where all the data are accurate to four decimal places. The updating interval length *T_n_* in the simulation is 1.0e−3 s, and the clock frequency of the FPGA is set to 10 ns.

The same simulation scenarios are calculated on a MATLAB R2007a platform with the strapdown algorithm in Section 2, the results are consistent with those shown in Table 1.

Figure 10 shows that the signal *GD_Rdy* has a pulse output at time 20.47 us, and the signal *OUT_Rdy* has a pulse output at time 30.66 us. This means that the start time and end time of the parallel strapdown algorithm on the FPGA platform are 20.47 us and 30.66 us, respectively. Then the execution time of this parallel strapdown algorithm is only 10.19 μs, when the clock frequency is selected as 10 ns. But the execution time of a strapdown algorithm on a DSP platform is generally in milliseconds. Thus the execution speed of parallel strapdown algorithm on the FPGA platform is much faster than the conventional algorithm on a DSP platform.

The resource utilization of the parallel strapdown algorithm on the hardware platform of the FPGA device XC6VLX550T is shown in Table 2 where Slice Registers, Slice LUTs and DSP48Es are the registers, the look-up tables and the multipliers based on the intellectual property (IP) hard-core of the Xilinx FPGA, respectively.

## 6. Conclusions

In this paper, a new generalized optimum strapdown algorithm with the coning and sculling compensation is presented, in which the PVA updating operations are carried out based on the single-speed structure in which all computations are executed at a single updating rate that is sufficiently high to accurately account for high frequency angular rate and acceleration rectification effects. Different from existing algorithms, the updating rates of the coning and sculling compensations are unrelated with the number of the gyro incremental angle samples and the number of the accelerometer incremental velocity samples. When the output sampling rate of inertial sensors remains constant, this algorithm allows increasing the updating rate of the coning and sculling compensation, yet with more numbers of gyro incremental angle and accelerometer incremental velocity in order to improve the accuracy of system. Then, in order to implement the new strapdown algorithm in a single chip FPGA, the parallelization of the algorithm is designed and its computational complexity is analyzed. The performance of the proposed parallel strapdown algorithm is tested on the software platform of Xilinx ISE 12.3 and the FPGA device XC6VLX550T hardware platform on the basis of some fighter data. It is shown that this parallel strapdown algorithm on the FPGA platform can greatly decrease the execution time of algorithm to meet the real-time and high precision requirements of system on the high dynamic environment, relative to the existing implemented on the DSP platform.

## Figures and Tables

**Table 1.** (**a**) Simulation results—gyro and accelerometer inputs; (**b**) Simulation results— attitude, velocity and position outputs.

**Sample time**	**Gyro incremental angle sample**			**Accelerometer incremental velocity sample**		
	**x axis (°)**	**y axis (°)**	**z axis (°)**	**x axis (m/s)**	**y axis (m/s)**	**z axis (m/s)**
t_n–1_	7.3154e−5	−4.0469e−6	7.0390e−6	−1.4102e−4	3.3985e−4	9.9255e−3
t_n_	7.1058e−5	3.9166e−6	7.8809e−6	−2.2112e−4	1.4560e−4	9.5916e−3
(a)						

**Update time**	**Attitude**			**Velocity**			**Position**		
	**roll angle (°)**	**pitch angle (°)**	**yaw angle (°)**	**x axis (m/s)**	**y axis (m/s)**	**z axis (ms)**	**latitude (°)**	**longitude (°)**	**altitude (m)**
t_n–1_	−0.1943	1.2738	121.8839	151.1450	−80.7500	9.3250	39.8598	116.4813	37.3570
t_n_	−58.1158	1.2720	−0.1906	151.1533	−80.7497	9.3103	39.8598	116.4813	37.3663
(b)									

**Table 2.** Resource utilization of parallel strapdown algorithm.

**Resource Type**	**Slice Registers**	**Slice LUTs**	**DSP8Es**
**Usage Amount**	24,837 (3%)	26,074 (7%)	105 (12%)
**Available Amount**	687,360	343,680	864
